# Integrating personalized gene expression profiles into predictive disease-associated gene pools

**DOI:** 10.1038/s41540-017-0009-0

**Published:** 2017-03-13

**Authors:** Jörg Menche, Emre Guney, Amitabh Sharma, Patrick J. Branigan, Matthew J. Loza, Frédéric Baribaud, Radu Dobrin, Albert-László Barabási

**Affiliations:** 1 Center for Complex Networks Research and Department of Physics, Northeastern University, Boston, MA 02115 USA; 2 Center for Network Science, Central European University, Budapest, 1051 Hungary; 3 CeMM Research Center for Molecular Medicine of the Austrian Academy of Sciences, Vienna, 1090 Austria; 4 Center for Cancer Systems Biology (CCSB) and Department of Cancer Biology, Dana-Farber Cancer Institute, Boston, MA 02215 USA; 5 Department of Medicine, Brigham and Womens Hospital, Harvard Medical School, Boston, MA 02115 USA; 6 Janssen Research & Development Inc., Spring House, PA 19477 USA

## Abstract

Gene expression data are routinely used to identify genes that *on average* exhibit different expression levels between a case and a control group. Yet, very few of such differentially expressed genes are detectably perturbed in individual patients. Here, we develop a framework to construct *personalized* perturbation profiles for individual subjects, identifying the set of genes that are significantly perturbed in each individual. This allows us to characterize the heterogeneity of the molecular manifestations of complex diseases by quantifying the expression-level similarities and differences among patients with the same phenotype. We show that despite the high heterogeneity of the individual perturbation profiles, patients with asthma, Parkinson and Huntington’s disease share a broadpool of sporadically disease-associated genes, and that individuals with statistically significant overlap with this pool have a 80–100% chance of being diagnosed with the disease. The developed framework opens up the possibility to apply gene expression data in the context of precision medicine, with important implications for biomarker identification, drug development, diagnosis and treatment.

## Molecular Medicine: Personalized expression profiles elucidate disease heterogeneity

A comparison of gene expression profiles of different patients with the same disease reveals a combinatorial model for disease heterogeneity. A framework is developed to generalize *group-wise* differential expression profiles to *personalized* perturbation profiles (PEEPs) for individual subjects. It is shown that similarities among patients with the same disease cannot be attributed to a few widely shared genes, but arise from more complex patterns of pairwise overlaps that often correspond to perturbations within the same biological pathway. This points to the existence of a *disease module*, i.e. a broader group of functionally related genes whose perturbations are associated with the specific disease. The overlap of an individual’s PEEP with the disease module accurately predicts disease status, suggesting their potential for developing combinatorial biomarker signatures or identifying most widely effective drug targets.

## Introduction

Microarray techniques, and more recently RNA sequencing have fundamentally changed our ability to explore the molecular mechanisms underlying complex diseases, being routinely used to identify disease-associated genome-wide changes in gene expression patterns. An important goal of these studies is the identification of differentially expressed (DE) genes, whose expression level systematically differs between a case (disease) and a control (healthy) group. The expectation is that such DE genes will help pinpoint the molecular processes perturbed in a disease, which in turn can be used as biomarkers for diagnosis and prognosis,^1, 2^ patient classification and drug target identification. For example differential expression patterns of whole blood cells have long been considered promising candidates for cheap, easily accessible biomarkers for multiple diseases.^3^

Despite their extraordinary use in research and medicine, the interpretation and validation of gene expression patterns continues to offer major challenges. Indeed, results from similar studies are often inconsistent, the proposed biomarkers are often not reproduced, and the identified DE genes rarely point to a unique set of disease-associated genes.^4^ For example, a meta study of multiple heart failure studies failed to identify any gene that is DE in all seven datasets, the most reproduced gene being DE only in four datasets^5^. Two main reasons are often listed as the source for these inconsistencies: (i) The comparison of different microarray-based measurements is hindered by important technical challenges, like the use of different platforms, dyes or statistical methods. (ii) There is intrinsic variability in gene expression levels, driven by both genetic factors, like the effect of single nucleotide polymorphisms and copy number variations on expression qualitative trait loci (eQTLs),^6, 7^ and non-genetic factors,^8–11^ arising from epigenetic modifications^12^ and the inherent stochasticity of biological processes.^13–15^ Here, we focus on a third important yet less explored factor: the heterogeneity of complex diseases, i.e., the possibility that multiple, only partially or non-overlapping molecular mechanisms can act in different patients with the same phenotype. For example, breast and colorectal tumors typically contain about 80 mutated genes.^16^ Yet, the mutations in different tumors have very little overlap, so that in only 22 tumors an astonishing total of more than 1700 mutated genes has been identified. To date, about 140 “driver genes” have been identified, whose mutation promotes tumorigenesis in most cancer types, but only two to eight of these driver genes are mutated in any individual tumor.^17^ A similar phenomenon is likely to occur at the gene expression level: many different perturbations may be associated with the same phenotype. We must therefore develop bottom-up methodologies that can interpret in a predictive fashion the inherent heterogeneity of individual perturbation profiles of both healthy and disease patients.

Here we introduce a framework to construct and integrate personalized perturbation profiles (PEEPs) from gene expression data, allowing us to systematically characterize the inherent heterogeneity of gene expression patterns. We test our approach on asthma, a chronic inflammatory disease of the lung and Parkinson’s disease (PD), a progressive disorder of the nervous system;^18^ and Huntington’s disease (HD), a neurodegenerative disorder caused by mutations in a single gene (Huntingtin).^19^ In all three diseases, we document a high heterogeneity between the PEEPs of individual patients. We show, however, that using a combinatorial model, these heterogeneous patterns can be integrated in a broad, yet highly predictive disease pool specific for each disease. Our results offer a conceptual change in the way we interpret disease-associated perturbations, in line with the emerging disease module hypothesis. Accordingly, disease-associated mutations perturb some cellular function that at the molecular level is encoded into a subnetwork of the underlying interactome. Therefore, multiple, often independent perturbations can impair the functional integrity of such a module, indicating that it is intrinsically impossible to associate a single gene or pathway to a specific pathophenotype.

## Results

**Fig. 1:**
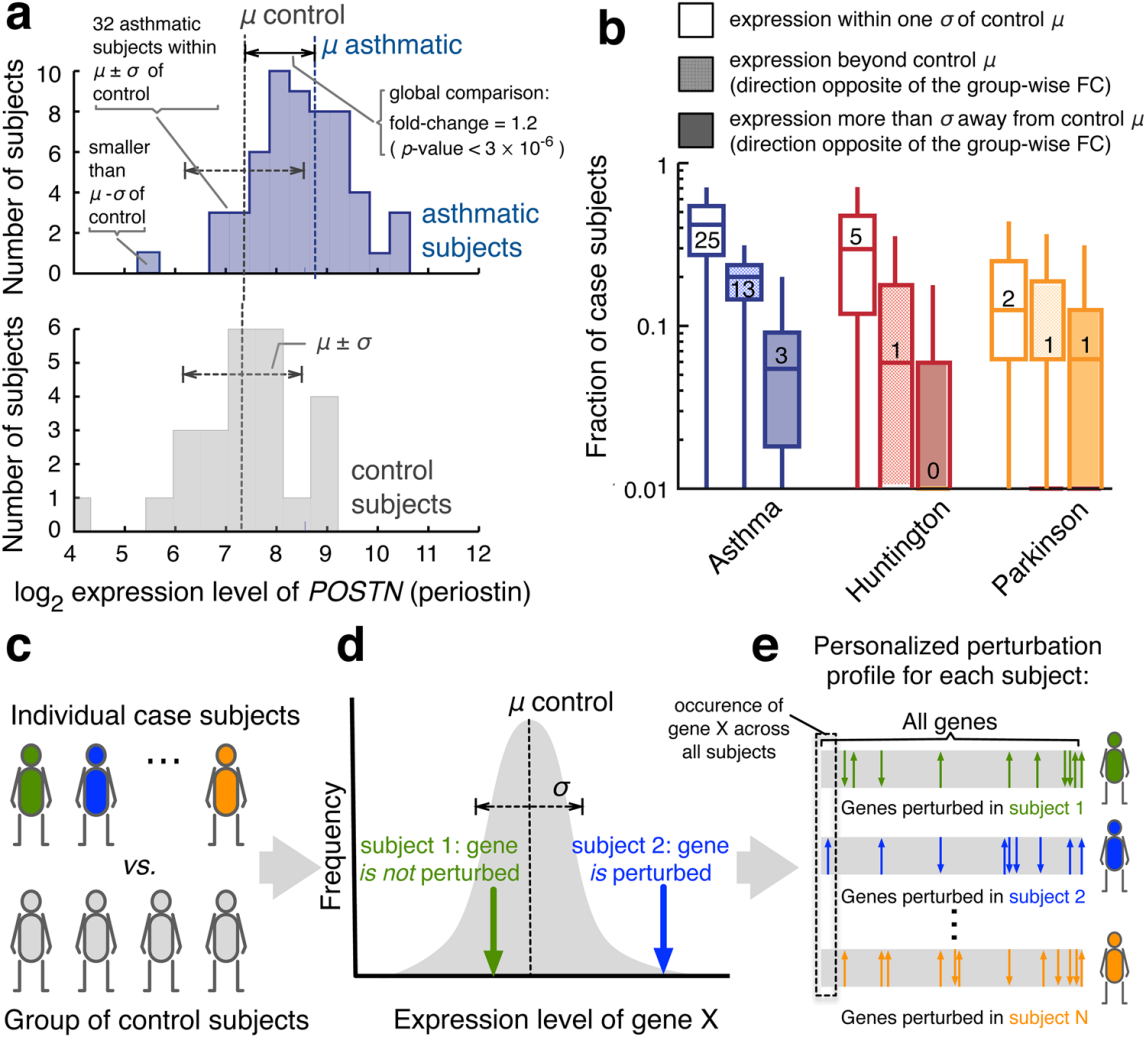
Personalized gene expression analysis. **a** Example of the distribution of expression levels for the asthma biomarker *POSTN*. While the group-based comparison (FC = 1.2, *p*-value <3 × 10^−6^) suggests a global up-regulation of *POSTN*, many asthmatic individuals exhibit normal or even down-regulated *POSTN* levels. **b** Fraction of case subjects in which genes that are denominated as being DE in a standard group-wise analysis display normal expression levels, or expression levels that suggest a dysregulation in the opposite direction. The distributions show the respective fractions over all group-wise DE genes. All whisker bars throughout this manuscript indicate the 5, 25, 50, 75, and 95th percentiles of the respective distributions. Small numbers within the bars indicate the absolute number of patients that the respective median fraction corresponds to. **c**–**e** Illustration of the proposed approach towards individual perturbation profiles: instead of comparing two groups of case and control subjects, we compare each case subject individually with the background of control subjects (**c**). Genes whose expression level is sufficiently far from the range observed in the control subjects **d** are denoted as perturbed in the respective individual. Together, the perturbed genes constitute a personalized, i.e. subject specific “barcode” (**e**)

To illustrate the inherent limitations of group-based differential expression analysis, consider the *POSTN* gene, coding for the protein periostin. Periostin is an established biomarker for asthma,^20–22^ its role in airway remodeling being exploited by an experimental asthma drug.^23^ The strong differential expression pattern between asthmatic and healthy subjects confirms its asthma association (Fig. 1a, fold-change FC = 1.2, *p*-value <3 × 10^−6^, Mann–Whitney *U*-test). Yet, while this group-wise difference is very pronounced, we find a more differentiated picture at the individual level: 25 out 55 asthmatic subjects have relatively low *POSTN* expression levels (within one standard deviation of the mean of the control group) and for 4 out of 25 control subjects, the *POSTN* level exceeds the mean level within the asthmatic group, violating the trend identified by the group-wise analysis. Overall, for 60% of asthmatic subjects, the expression level of *POSTN* is within one standard deviation of the mean of the control subjects, indicating that genes that show systematic expression level differences between groups are not up-regulated or down-regulated in each individual with the phenotype.

To generalize the above observations, we inspected the expression levels of all genes that were DE according to a standard group-wise analysis in asthma, PD and HD (see Methods). As shown in Fig. 1b, 13, 30 and 42% of all case subjects for HD, PD and asthma, respectively, exhibit an expression level that is compatible with random expectation for control subjects (within one standard deviation *σ* of the mean control level *µ*). Furthermore, 6, 7 and 20% of all case subjects have expression levels that were beyond the control mean in a direction that is the opposite to the one suggested by the group-wise difference. We presume that the effect is strongest in asthma due to the larger population sizes in the respective dataset.

### A framework for personalized gene-expression analysis

We can overcome the limitation of group-wise methods that only detect mean changes between two groups and turn individual expression heterogeneity into a predictive information by constructing personalized perturbation profiles that reflect expression changes within a single subject (Fig. 1c). For each gene *i* of subject *j,* we compare the expression level ${l}_{i}^{j}$ to the reference distribution of expression levels of that gene within the control group (Fig. 1d). The deviation is measured by the *z*-score

$${z}_{i}^{j}=\frac{{l}_{i}^{j}-{\langle {l}_{i}\rangle }_{{\rm{cont}}}}{{\sigma }_{{\rm{cont}}}({l}_{i})},$$

capturing how many standard deviations ${\sigma }_{{\rm{cont}}}({l}_{i})$ the individual expression level ${l}_{i}^{j}$ deviates from the mean value ${\langle {l}_{i}\rangle }_{{\rm{cont}}}$ of the control group. We then use a threshold *z*_thresh_ to identify the genes that are sufficiently perturbed in an individual subject. The resulting individual perturbation expression profile (PEEP) of a subject can be viewed as a “barcode”, representing the genes that are up-regulated (${z}_{i}^{j}  >{z}_{{\rm{thresh}}}$) or down-regulated (${z}_{i}^{j} < -{Z}_{{\rm{thresh}}}$) compared to the control group (Fig. 1e). In the following, we focus on profiles obtained for *z*_thresh_ = 2.5 (see Supplementary Fig. 1 for the impact of *z*_thresh_ on our results).

**Fig. 2:**
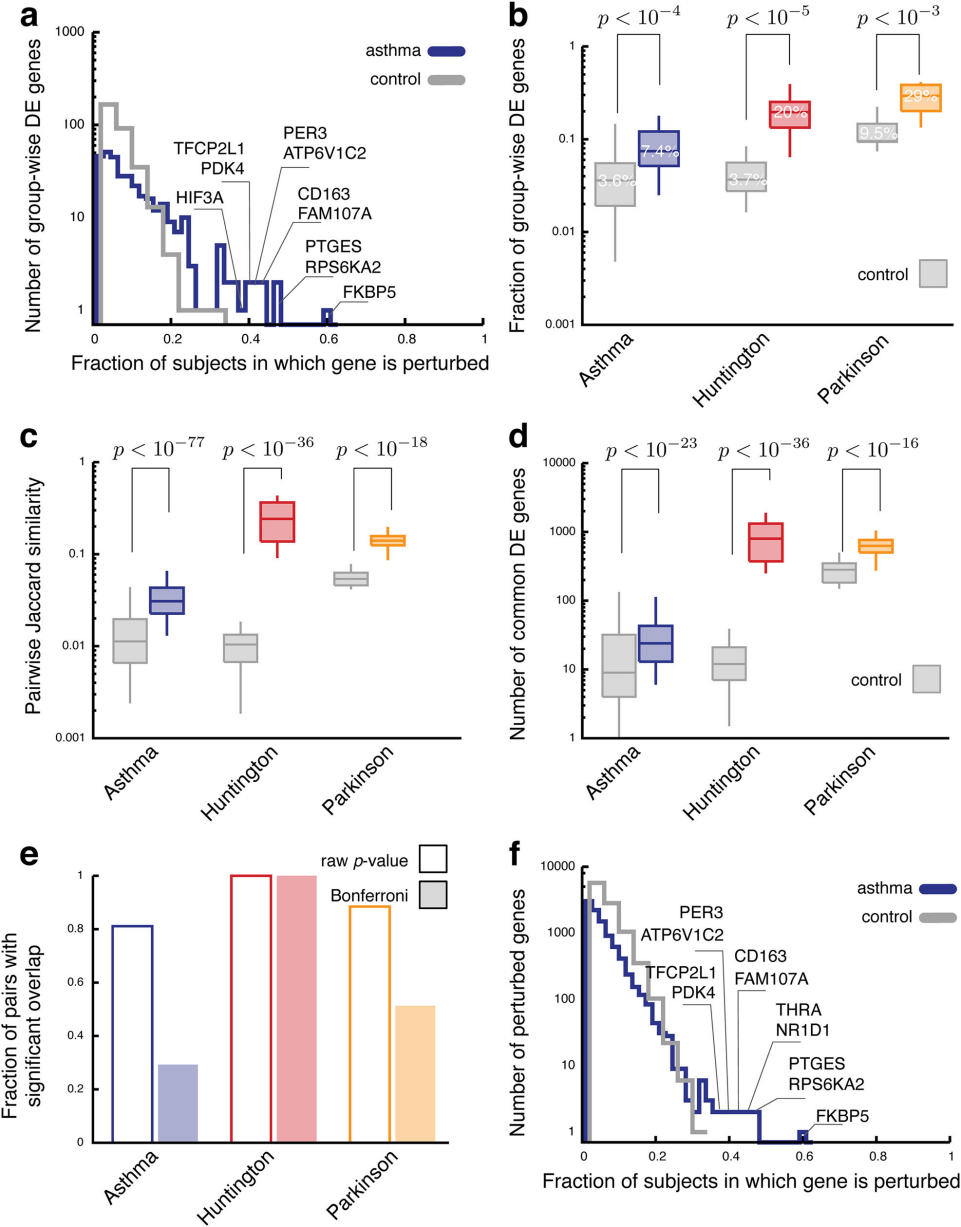
Heterogeneity among the PEEPs. **a** Distribution of the fraction of all PEEPs in which a gene appears that has been identified in a standard group-wise analysis (for asthma). **b** Fraction all group-wise DE genes found in the PEEPs for asthma patients. **c**, **d** Pairwise overlap of the genes in the PEEPs as measured by the Jaccard index (**c**) and the number of common genes (**d**). **e** Fraction of all case subject pairs whose gene overlap is statistically significant (Fishers’s exact test, *p*-value <0.05). **f** Distribution of the fraction of asthma patient PEEPs in which a gene appears

To characterize the PEEPs, we compare the group of genes perturbed in individuals with DE genes obtained from a standard group-wise approach (see Methods). Our first observation is that only for HD, we find group-wise DE genes that are contained in all individual profiles. For asthma and PD, no single gene is perturbed in all case subjects. Figure 2a shows the distribution of the number of subjects whose personalized profile includes the same gene for asthma (see Supplementary Fig. 3 for HD and PD). The maximal number of subjects sharing the group-wise DE gene *FKBP5* is 33 out of 55, i.e., 60% of all asthmatic subjects. The mean number of asthmatic subjects in which a group-wise DE gene is significantly perturbed is 6, or 11% of all asthmatic subjects. In PD, there is one group-wise DE gene that is shared among 15 out of 16 case subjects, in HD there are 18 genes shared among all 17 patients. On average, the group-wise DE genes are contained in 31 and 29% of the case subjects for PD and HD, respectively (see also Supplementary Fig. 3). Figure 2b summarizes the fraction of the group-wise DE genes contained in the individual profiles. While this fraction is significantly higher in case subjects than in control subjects, it is still surprisingly low: For asthma, on average less than 8% of the group-wise DE genes are found in an individual profile. The highest numbers are observed for PD, where case subjects contain on average 29%. These results lead to two key main findings, on one end indicating that often DE genes identified by standard group-wise approaches are significantly perturbed only in a small fraction of individuals with the disease and likewise, any individual displays only a small fraction of all group-wise DE genes in their PEEP.

### Quantifying the heterogeneity among individual perturbation profiles

To quantify the underlying expression heterogeneity of a disease, we move beyond the group-wise DE genes, and ask instead how similar are the PEEPs of two individuals with the same disease. Figure 2c shows the distribution of Jaccard indices $J=\frac{|A\cap B|}{|A\cup B|}$ for all pairwise gene sets A and B of the individuals in the case, and control groups of three diseases. For asthma, the mean pairwise similarity $(\langle J\rangle =3\times {10}^{-2})$ is three times higher in the case group than in the control group $(\langle J\rangle =1\times {10}^{-2})$. While this difference is highly significant (*p-*value <10^−77^, Mann–Whitney *U* test), in absolute numbers the overlap is small: while a typical asthmatic subject has on average 379 perturbed genes, the average number of shared perturbed genes between two asthmatic subjects is only 24 (Fig. 2d). For HD and PD, the average overlap between the profiles of two cases is much higher (796 and 627 common genes, respectively) due to the much higher number of genes in the individual perturbation profiles. Yet, the Jaccard similarities remain relatively small, observing $\langle J\rangle \sim 0.24$ and $\langle J\rangle \sim 0.14$ for HD and PD, respectively. The same analysis can also be performed on the full continuous *z*-score profiles using Pearson correlation as measure of similarity, yielding similar results (see Supplementary Fig. 2)

To quantify whether the observed overlap between the PEEPs of the case subjects could have emerged by chance, we calculated the statistical significance for each pair individually using Fisher’s exact test. As expected, we find the overlap to be significant for most subject pairs, even after applying the most conservative Bonferroni correction (Fig. 2e).

The significant pairwise overlap documented in Fig. 2c–e is not the result of a set of genes that are common to most subjects. Indeed, as shown in Fig. 2f for asthma, most genes within the individual profiles are perturbed only in relatively few individuals, the mean number of subjects being 3, which is 5% of all the subjects (see Supplementary Fig. 3 for HD and PD). The most frequently perturbed gene appears in the PEEP of 33 subjects, representing 60% of the case cohort. Comparing Fig. 2a and f, we notice that the genes appearing in many subjects’ PEEPs are often also identified in the group-wise analysis, which is expected. Yet, the lack of genes present in all individual perturbation profiles again illustrates that a group-wise analysis offers only a partial picture of the expression patterns that characterize complex diseases.

This leads to our second main result: we observe highly significant similarities between the PEEPs of case subjects, similarities that are absent in healthy subjects. These similarities cannot be attributed to a few widely shared DE genes identified by the group-wise differential expression analysis, but arise from more complex patterns of pairwise overlaps.

### Functional analysis of the perturbation profiles

**Fig. 3:**
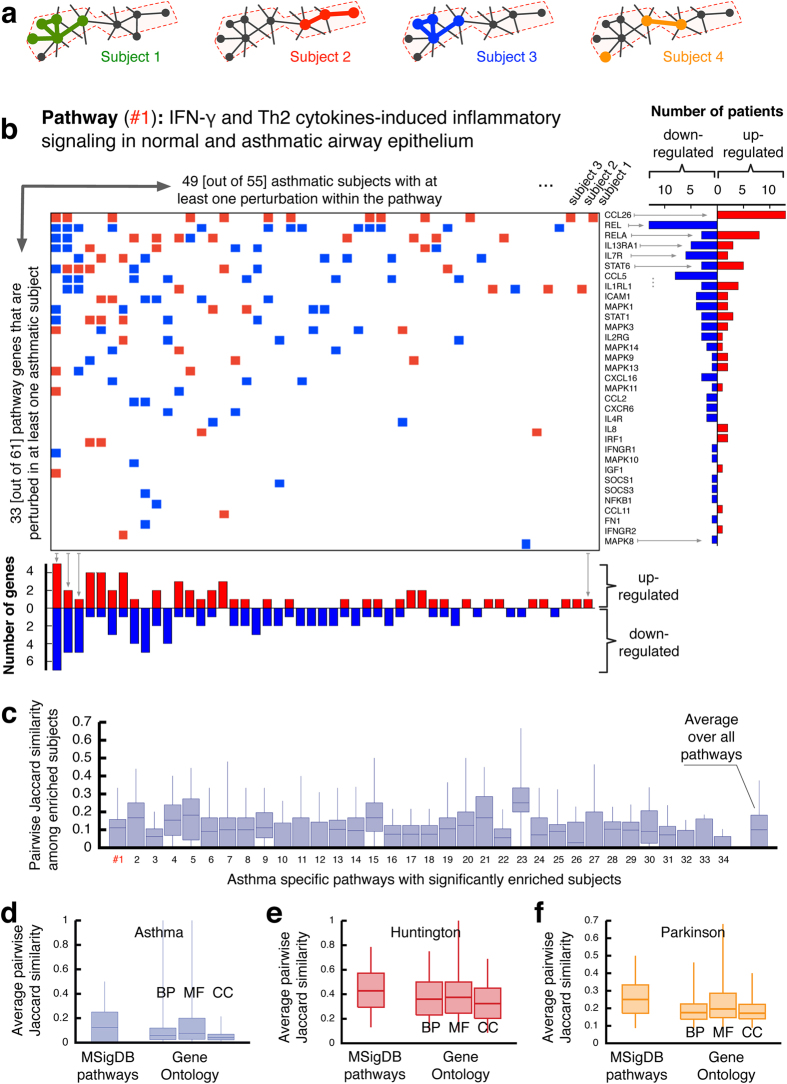
Functional characteristics of the genes in PEEPs. **a** A schematic figure illustrating how the same pathway associated with a specific function may be disrupted by perturbations at different locations in different subjects. **b** Individual perturbations of all asthmatic subjects within the asthma-specific pathway IFN-γ and Th2 cytokines-induced inflammatory signaling in normal and asthmatic airway epithelium. Each *row* corresponds to one pathway gene and each *column* to one subject. On the *right:* the number of subjects that have the respective gene up-regulated or down-regulated. *Below*: number of up-regulated or down-regulated genes within the pathway for each subject. **c** Pairwise similarity as measured by the Jaccard index of the pathway perturbations of all subject pairs whose profiles are significantly enriched within the pathway (Fisher’s exact test with Bonferroni correction, *p*-value *<*0.05) for all considered asthma-specific pathways (see Supplementary Table 1). Note that only the genes within the respective pathway areused for the comparison. **d-f** as in (**c**), but averaged over all geneGO terms and general MSigDB pathways that are significantly enriched in the profiles of the respective subjects (see Methods). *BP* biological process, *MF* molecular function, *CC* cellular component

The low overlap between the personalized profiles of case subjects prompts us to ask how the molecular level heterogeneity translates into relatively homogeneous disease phenotypes. To address this, we examine the extent to which the individual profiles reflect disruptions in common disease-specific pathways (Fig. 3a). We compiled a list of 35 previously identified asthma-related pathways from GeneGo (portal.genego.com) (Supplementary Table 1), and compared the individual perturbation profiles of each asthma subject with each pathway. Almost all pathways show at least one perturbation in most subjects, and all pathways are significantly enriched in at least two individuals (Fisher’s exact test, *p*-value <0.05, Bonferroni correction for number of pathways). Take for example the pathway IFN-γ and Th2 cytokines-induced inflammatory signaling in normal and asthmatic airway epithelium, in which 49 out of 55 asthma subjects (89%) have one or more PEEP perturbation. Yet, as Fig. 3b shows, the precise location of the perturbations within the pathway varies considerably between the individuals. In total, 33 (out of 61) genes of the pathway are up-regulated or down-regulated in one or more patients. The genes that appear most frequently (13 subjects) are *CCL26* and *REL*, both previously associated with asthma.^24–26^ These two genes are also consistently perturbed in the same direction (Fig. 3b). At the same time, several genes, like *IL13RA1* or *STAT6*, are up-regulated in some patients, and down-regulated in others, suggesting that for these genes, the direction of the perturbation is secondary for the disease association. A possible biological interpretation could be that these genes correspond to tightly regulated checkpoints within the pathway, such that any deviation from the homeostatic level would result in a disease-associated perturbation, regardless of the direction of the deviation.

We next determined the Jaccard similarity of the respective individual perturbed pathway genes for each pair of subjects, whose PEEPs are significantly enriched with genes of the pathway. The low similarity values (*J *∼ 0.1, Fig. 3c) confirm that although all considered subjects show significant perturbations in these asthma-specific pathways, the specific perturbations differ greatly between subjects. These differences limit the power of group-wise DE gene sets to detect affected pathways. As shown in Supplementary Table 1, group-wise DE genes cover only a small fraction of the asthma-related pathways: only 7 out of 35 pathways show nominally significant enrichment (uncorrected *p*-value *<*0.05, Fisher’s exact test); after Bonferroni correction, only two pathways remain. Taking individual perturbation profiles into account thus considerably boosts the ability of enrichment analysis tools to identify important disease-associated pathways.

We repeated the analysis of the heterogeneity among perturbed pathways also for AD and PD, using general pathway annotations from Molecular Signatures Database (MSigDB)^27^ and functional gene ontology (GO).^28^ The results again indicate that the same biological function or pathway is perturbed in different ways in different patients (Fig. 3d–f). These results allow us to formulate our third main result: while patients show considerable perturbation heterogeneity at the PEEP level, they show a high degree of homogeneity at the pathway level. In other words, the different perturbations within a certain molecular pathway lead to similar outcomes, in line with the disease module hypothesis.

### Predicting diseases from PEEPs

Taken together, our results indicate that patients with the same disease exhibit highly heterogeneous perturbations that nevertheless point towards common functional disruptions. This suggests the existence of a broader group of genes, whose perturbations are associated with the specific disease. As we demonstrate next, by compiling all genes that are perturbed in a significant fraction of the case subjects, we can accurately predict the disease state of each patient.

**Fig. 4:**
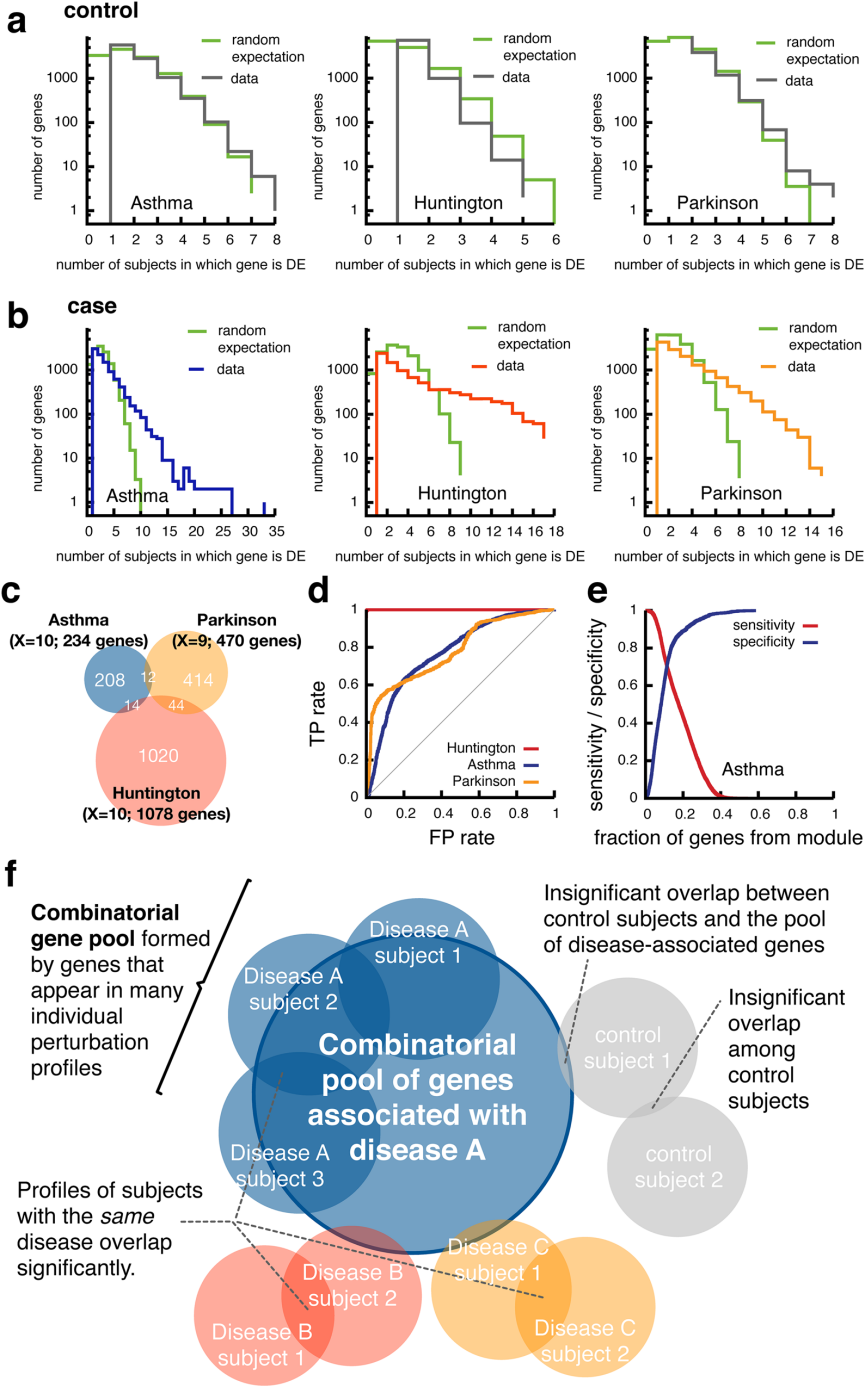
Integrating the personalized profiles into a predictive pool of disease-associated genes. **a**, **b** Distribution of the number of individual perturbation profiles in which a gene appears for **a** control and **b** case subjects of the three considered diseases. The two curves in each panel correspond to the actual data and the random expectation according to a model of randomly selected genes (*green*). **c** Venn diagram of three broad gene pools compiled from genes that are in at least *X* individual perturbation profiles. **d** The ROC for the disease state classification by the fraction of the broad gene pool that is contained in a subject’s perturbation profile. The AUC values are 0.77 ± 0.03, 0.81 ± 0.06 and 1.0 ± 0.0 for asthma, PD and HD, respectively (mean value ± standard deviation computed over 100 cross-validations). **e** Sensitivity and specificity as a function of the fraction of broad gene pool for asthma (*z*_thresh_ = 2.5; *X* = 10). **f** Illustration of the disease model suggested by the analysis of PEEPs

Given the relatively high number of genes perturbed in the individual profiles (Fig. 2a), a gene may appear in several subjects simply by chance. Indeed, we find that the number of genes that are shared among control subjects is compatible with random expectation (Fig. 4a). In the healthy control group, possible individual perturbations of the regulatory network are unlikely to be shared among different individuals. For this group, the simplified model of complete independence between subjects is thus a reasonable approximation, as also shown by the good agreement between data and theory reported in Fig. 4a. For case subjects, however, the number of shared genes significantly exceeds the random expectation (Fig. 4b). These frequently appearing genes point to the existence of a disease module, a pool of genes whose perturbations are often associated with the disease. Using a combinatorial model, whose basic assumption is that the individual PEEPs constitute random subsets of the disease module (see Methods for details), we can determine the size of this module analytically, obtaining gene pools containing 234 genes for asthma, 470 for PD and 1076 for HD (Fig. 4c).

Perturbations of these modules uniquely characterize the respective diseases. To show this, we used a repeated cross-validation approach, and determined the different PEEP’s overlap with the disease module (see Methods). We find that the fraction of genes from the disease module perturbed in an individual subject accurately predicts whether the subject has the disease. For asthma, the PEEPs of case subjects contain on average 21% of the asthma disease pool, compared to less than 7% for the control subjects. For PD and HD, the overlap of the case subjects with the corresponding disease modules is much higher, obtaining 65 and 86% respectively, compared to 20 and 6% for the control subjects. This indicates that PD and HD are characterized by a more specific set of characteristic perturbations, while asthma displays a more heterogeneous range of associated perturbations. The receiver operating characteristics (ROC) in Fig. 4d show that the fraction of genes from the general pool that are contained in an individual’s perturbation profile can be used as a near highly accurate classifier to distinguish between case and control subjects with high sensitivity and specificity (Fig. 4e). The area under the curve (AUC) values for asthma, PD and HD are 0.77 ± 0.03, 0.81 ± 0.06 and 1.0 ± 0.0 (mean value ± standard deviation computed over 100 cross-validations), respectively. Note that these results were obtained with the threshold *z*_thresh_ = 2.5 that we used throughout the manuscript, and can be further improved by optimizing *z*_thresh_ and the minimal number of PEEPS *X* in which a gene must appear to be considered for the disease pool (Supplementary Fig. 4). We also benchmarked our results against a widely used *k*-nearest neighbor (knn) classification algorithm^29^ (Methods), and found comparable performance (AUC values of 0.80 ± 0.03, 0.85 ± 0.06 and 0.98 ± 0.02 for asthma, PD and HD, see Supplementary Fig. 5). This not only demonstrates that a classifier based on our combinatorial model offers predictive power similar to the one of state-of-the-art machine learning approaches, but more generally confirms the validity and self-consistency of the basic PEEP concept itself. Indeed, the PEEP concept complements exiting machine-learning approaches as it offers a straightforward biological interpretation of the obtained classification in terms of overlapping perturbation profiles that can also easily be further investigated, using for example, gene set enrichment analyses as demonstrated above. Furthermore, the PEEP-based classification procedure directly yields a measure for the heterogeneity of the disease, as the combinatorial model explicitly uses the overlap of an individuals PEEP with the broad disease pool to classify the disease status.

## Discussion

Group-wise expression analysis has two important limitations: (i) It can only identify genes that are consistently (i.e., in the same direction) perturbed in a large fraction of the patients. (ii) It does not yield patient-specific information. Here, we introduced a simple, yet powerful method that overcomes these limitations and offers personalized perturbation profiles (PEEPs). The method can be interpreted as a generalization of group-wise differential expression methods with PEEPs representing personalized DE genes. As a consequence, the PEEPs can be easily interpreted, and further analyzed using established tools, such as the gene-set enrichment analysis used above.

As illustrated in Fig. 4f, the overlap between the genes perturbed in any two patients is relatively small. Indeed, of the three diseases considered here, only HD exhibited genes that were perturbed in all case subjects, likely rooted in the fact that HD is a classic monogenic disease. For asthma and PD, on the other hand, there is not a single gene expressed in the PEEP of all patients.

Despite the high gene level variability, the commonalities at the functional and pathway level indicate that complex diseases arise from disruptions of certain biological processes or disease modules,^30^ hence the observed heterogeneity simply reflects the molecular diversity of such disruptions. We therefore expect considerable variability among the expression profiles of subjects with the same disease not despite, but because they all have the same disease. Recently, a number of studies proposed various strategies for dissecting disease heterogeneity, in particular in the field of cancer. The PARADIGM algorithm,^31^ for example, infers patient-specific pathways using various omics-type information, such as expression and mutational data, together with curated pathway interactions. Another widespread algorithm, HotNet2^32^ tackles the genetic heterogeneity of different cancer samples using the concept of information propagation starting from known mutations, in order to identify cancer-related subnetworks in signaling networks. In this work, we document the existence of large disease module also on other disease areas, and using transcriptional data only. We find that a sufficient level of random perturbations among these disease modules can accurately predict the presence/absence of a particular disease. We integrated the personalized profiles of all patients to reconstruct the respective disease module, finding that the fraction of genes in an individual’s PEEP is a near perfect predictor for a patient’s disease status. This suggests that personalized profiles could identify combinatorial biomarker signatures that go beyond single markers. With next-generation sequencing technology advancing at a fast pace, there is great potential for applying RNAseq technologies to identify transcriptional signatures also in a clinical setting.^33^ Such signatures are of key importance for personalized medicine and could, for example, help diagnose previously unrecognized diseases. While the results presented here provide first evidence of the general feasibility of using our approach to obtain predictive biomarkers, a comprehensive reference base across all relevant diseases, and more extensive tests concerning the robustness and reliability of the resulting disease pools will be required towards an actual clinical application. The observed heterogeneity among the individual perturbation profiles further indicates that single-target drugs may be effective only in a small number of patients. Instead, multi-target strategies may prove more promising for drug development.^34^ Our approach can be used to quantitatively assess the expected fraction of patients for which a drug is expected to be effective, helping guide the development of targets with maximal efficacy.

## Methods and Materials

### Gene expression data

We use data from an ongoing study by Janssen Research & Development for asthma (manuscript in preparation), and previously published expression profiling studies for HD^19^ and idiopathic Parkinson’s disease.^35^ The asthma dataset contains 55 case subjects with moderate or severe asthma, and 25 gender-matched and age-matched healthy control subjects, see ref. 36 for a detailed description of the cohort. The asthma samples were collected from bronchoscopy (endobronchial biopsies and brushings), preserved immediately in RNAlater^®^ solution and then maintained at −70 °C. Qiagen miRNeasy kit (Qiagen; Germantown, MD, USA) and NuGen ovation pico WTA kit (NuGen Technologies; San Carlos, CA, USA) were used to extract and amplify RNA. cDNA is profiled using Affymetrix HG-U133+PM chip (Affymetrix, Santa Clara, CA, USA). CEL files were assessed using Almac Diagnostics Microarray Toolbox for quality control (chip image analysis, Affymetrix GeneChip QC, RNA degradation analysis, distribution analysis, principal components analysis, and correlation analysis) and technical outliers are excluded. Robust multi-array (RMA) method is used to renormalize the profiles, followed by batch effect adjustment via linear modeling of batch (as random factor) and cohort. The HD dataset^19^ (GEO accession number GSE1767) contains analysis of blood samples from 17 case subjects (5 presymptomatic and 12 symptomatic) and 14 control subjects. In HD, the gene expression is suggested to be altered in a variety of tissues including peripheral blood. Affymetrix U133A GeneChips, and Amersham Biosciences CodeLink Uniset Human I and II bioarrays were used to analyze the gene expression in blood samples. The Parkinson’s disease data (GSE7621) contain 16 case and 9 control subjects for which multiregional gene expression analysis was conducted in postmortem brain using Affymetrix HG U133 Plus 2.0 gene chips. For the PD and HD datasets, the details of the sample generation and expression profiling can be found in the original publications. We reprocessed the raw data set in GEO for Parkinson’s using RMA with quantile normalization as implemented in the R package ‘affy’. We verified the quality of the data sets by checking the gene expression distribution and sample clustering in PCA. All expression levels in the PD and HD data were log_2_ -transformed to facilitate direct comparison of the three data, overall results do not depend on the transformation, however. Basic statistics of the used datasets are shown in Supplementary Fig. 1.

### Group-wise differential expression analysis

We identify the genes DE between case and control subjects using the limma R Bioconductor package.^37^ The difference between expression levels of case and control subjects are assessed by fitting the expression levels to a linear model using one coefficient for each group in the design matrix. The probe-sets were mapped to Entrez Gene IDs using the platform annotation files in each data set. In case there were multiple probe sets corresponding to the same Gene ID, the probeset with the maximum expression was used in the analysis. The *p*-values were corrected for multiple hypothesis testing using the Benjamini–Hochberg method.^38^ At a cut-off of FDR *<*0.2, we obtain 417, 524 and 7419 DE genes for asthma, PD and HD, respectively.

### Personalized perturbation analysis

To construct the personalized perturbation profile of a subject *j*, we compare the expression level ${l}_{i}^{j}$ of each of its genes *i* to the reference distribution of expression levels of the same gene within the control group. The extent to which gene *i* is perturbed in subject *j* is quantified by the *z*-score ${z}_{i}^{j}=\frac{{l}_{i}^{j}-\langle {l}_{i}\rangle {\rm{cont}}}{{\sigma }_{{\rm{cont}}}({l}_{i})}$ that indicates by how many standard deviations ${\sigma }_{{\rm{cont}}}({l}_{i})$, the individual expression level ${l}_{i}^{j}$ is away from the mean value $\langle {l}_{i}\rangle$ of the control group. Note that if subject *j* itself is part of the control group, we do not consider it for the computation of the reference distribution but use only the remaining control subjects. We then use a threshold *z*_thresh_ to define the set of genes that are perturbed in an individual subject. Positive *z*-scores ${z}_{i}^{j}  >{z}_{{\rm{thresh}}}$ indicate up-regulation, negative values ${z}_{i}^{j} < -{Z}_{{\rm{thresh}}}$ indicate down-regulation. The role of *z*_thresh_ is thus analogous to the one of cutoffs for calling differential expression in standard group-wise analyses. Higher *z*_thresh_ values result in smaller, more stringent PEEPs that potentially miss out relevant genes with less pronounced perturbations, while lower *z*_thresh_ values provide a more global picture that may, however, also contain an increased number of false positives. The precise choice of *z*_thresh_ can be optimized for specific purposes, such as disease patient classification (compare with Supplementary Fig. 4).

We systematically evaluated the stability of the obtained *z*-scores against changes in the population size by removing increasing numbers of subjects from the control population: we first calculated the expression level that corresponds to *z*-score = 2.5, compared to the average mean and standard deviation of all genes and all control subjects. We then observed how the *z*-score of this expression level changes while randomly removing an increasing number of subjects from the pool of control subjects (up to 50% of the original population). For each gene, we then calculated the ratio of the *z*-score obtained from the decreased population to the original *z*-score calculated from the original population. Supplementary Fig. 6 shows that for small numbers of the removed subjects, the fluctuations are very small, indicating stable *z*-scores. As expected, the fluctuations grow as more and more subjects are removed. We conclude that all considered datasets have a sufficient number of control subjects in order to yield reliable perturbation profiles. Conversely, the data suggest that increasing population sizes can further stabilize the PEEPs, ultimately converging into a fixed pool of disease-specific genes.

### Analytical comparison with randomly distributed genes

To determine the minimal number *X* of case subjects in which a gene must be perturbed in order to be collected into the global combinatorial pool of disease associated genes, we use a comparison with random expectation. We consider a null model where each subject has *g* perturbed genes that are drawn completely at random from all *G* genes. The probability for one gene to be perturbed in exactly *k* out of *n* subjects is then given by the binomial distribution

$$f(k;n,p)=\Pr (x=k)=\left({n\atop k}\right){p}^{k}{(1-p)}^{n-k}$$

with *p* = *g/G*. Using the mean number of genes observed in the individual profiles for *g*, the histogram of the number of subjects per gene can now be obtained by simply multiplying$G\times f(k;n,p)$. We find excellent agreement between this formula and the distributions observed among the control subjects, but, as expected, not for case subjects (Fig. 4a, b). The maximal number of subjects *X*_rand_ that are expected per gene according to this random model can be obtained from

$${\sum _{k={X}_{{\rm{rand}}}}^{n}}G\,f(k;n,p) < 1,\,$$

which can be solved by simply testing the increasing values. Finally, we choose the minimal value as *X* = *X*_rand_ + 1, thereby ensuring a broad, yet high-quality pool of disease-associated genes. The calculated values are *X* = 10 for asthma, *X* = 9 for PD and *X* = 10 for HD.

### Cross-validation analysis for disease state prediction

We performed a fivefold cross-validation analysis using the fraction of genes of the combinatorial pool of disease-associated genes that is contained in a subject’s personal perturbation profile to predict the disease state of the subject. Note that, we do not take the direction of the perturbation into account. If the fraction is larger than a given threshold that can be determined from the training data we classify the subject as ‘case’, otherwise as ‘control. This threshold not only allows for patient classification, but can also be interpreted as a direct measure of the heterogeneity of a disease.

For the cross-validation, we randomly split the subjects into five groups having similar proportions of cases and controls as in the full dataset. We then iteratively use each group as the validation set, and the remaining four groups as training data to generate the PEEPs and the combinatorial disease pool. Next, we calculate the fraction of the combinatorial pool that is contained in the PEEP of each subject in the validation set. By using all identified fractions as putative thresholds for classification as ‘case’ or ‘control’ and comparing with the true labels, we then construct the ROC curve and calculate the AUC. Note that the classifier is completely blind to the information of the left-out validation subjects, thus avoiding overfitting due to the fact that the combinatorial pool itself is compiled from all genes that are perturbed in *X* or more case subjects. The entire procedure is repeated 100 times to get robust estimates of the ROC curve and the AUC.

We further compared the performance of the PEEP-based classification to a knn-based classification. For every sample in the test set, we calculated the gene expression correlation with all samples in the training set, and then ranked the training samples according to the strength of the correlation. The known disease states of the *k* most similar samples (i.e., highest correlation) is then used to score the test sample’s likelihood to belong to the same class. After evaluating a range of values of *k* (=3, 5, 10, 15, 20), we found *k* = 15 to offer the highest prediction accuracy. Note that while the knn method allows for a high-quality classification, the subsequent interpretation of a classification result is less straightforward compared to the PEEP approach above, which is directly based on overlapping gene sets that can be immediately further investigated and potentially validated.

To estimate the influence of the sample size on the final accuracy of the classification analysis, we further repeated both the knn and the PEEP-based classification using a twofold validation scheme, such that only half of the case and control subjects are available for training. The results shown in Supplementary Fig. 5 demonstrate that both approaches are rather robust against variations in the sample size (AUC values for the PEEP-based approach are 0.72, 0.78 and 1.0 for asthma, PD and HD, respectively).

### Functional gene annotation data

To analyze the biological function of genes and gene sets we use GO terms, general pathway annotations and asthma-specific pathways. GO annotations^28^ were downloaded from http://www.geneontology.org/. We only use high confidence annotations associated with the evidence codes EXP, IDA, IMP, IGI, IEP, ISS, ISA, ISM or ISO and further remove all associations with a non-empty “qualifier” column.^39^ Since the provided GO files only contain the most specific annotations explicitly, we add all implicit more general annotations by up-propagating the given annotations along the full GO tree.

The general pathway annotations were taken from the MSigDB published by the Broad Institute, Version 4.0.^27^ MSigDB integrates several pathway databases; we use those from KEGG, Biocarta, and Reactome.

Asthma-specific pathways (Supplementary Table 1) were compiled using the GeneGo Software.

### Gene set enrichment analysis

The enrichment analysis between a given gene set and a pathway or GO annotation (‘term’) is done using Fisher’s exact test. We considered a term to be significantly enriched if *p*-value *<*0.05 (Bonferroni correction for number of tested terms). For each bar in Fig. 3d–f, we first determined all terms that are significantly associated with the genes in the individual profile of at least three case subjects. For each significant term, we then computed the Jaccard index for all possible pairs of subjects with profiles enriched with the respective term. Note that we use only the genes associated with the respective term to compute the Jaccard index. Finally, we combine all Jaccard values of all pairs and all GO terms into one distribution, which is represented by the whisker bars.

### R-package

We provide the R package ‘PePPeR’ (Personalized Perturbation ProfileR), which includes functions to fetch expression data sets from the GEO database, identify group-wise DE genes and construct individual perturbation profiles. The R package along with its documentation is available at https://github.com/emreg00/pepper.

## Electronic supplementary material

Supplementary Figure S1

Supplementary Figure S2

Supplementary Figure S3

Supplementary Figure S4

Supplementary Figure S5

Supplementary Figure S6

Supplementary Table 1
